# The CPR outcomes of online medical video instruction versus on-scene medical instruction using simulated cardiac arrest stations

**DOI:** 10.1186/s12873-016-0092-3

**Published:** 2016-07-12

**Authors:** Chaiyaporn Yuksen, Sorravit Sawatmongkornkul, Jarupol Tuangsirisup, Kittisak Sawanyawisuth, Yuwares Sittichanbuncha

**Affiliations:** Department of Emergency Medicine, Faculty of Medicine, Ramathibodi Hospital, Mahidol University, Bangkok, 10400 Thailand; Department of Medicine, Faculty of Medicine, Khon Kaen University, Khon Kaen, 40002 Thailand; The Research Center in Back, Neck Other Joint Pain and Human Performance (BNOJPH), Khon Kaen University, Khon Kaen, 40002 Thailand

**Keywords:** Ventricular tachycardia, Pulseless electrical activity, CPR, Online, On-scene

## Abstract

**Background:**

Non-traumatic cardiac arrest is a fatal emergency condition. Its survival rate and outcomes may be better with quick and effective cardiopulmonary resuscitation (CPR). Telemedicine such as telephone or real time video has been shown to improve chest compression procedures. There are limited data on the effects of telemedicine in cardiac arrest situations in the literature particularly in Asian settings.

**Methods:**

This study was conducted by using two simulated cardiac arrest stations during the 2014 annual Thai national conference in emergency medicine. These two stations, nos. 5 and 11, were a part of the conference activity called “EMS rally” which was comprised of 14 stations. Both stations were shockable and out-of-hospital cardiac arrest situations; station 5 was online instructed, while station 11 was on-scene instructed. There were 14 representative teams from each province from all over Thailand who participated in the rally. Each team had one physician, one nurse, and two emergency medicine technicians. Eight CPR outcomes were evaluated and compared between the online versus on-scene situations.

**Results:**

There were 14 representative teams that participated in the study; a total of 14 physicians, 14 nurses, and 28 emergency medicine technicians. The average ages of participants in all three occupations were between the second and third decade of life. The percentages of participants with more than 3 years in ambulance experience was 7.1, 64.3, and 53.6 % in the physicians, nurses, and EMTs groups. The median times of all outcomes were significantly longer in the online group than the on-scene group including times from start to chest compression (total 102 vs 36 s), total times from the start to VT/VF detection (187 vs 99 s); times from VT/VF detection to the first defibrillation (57 vs 28 s); and times from the start of adrenaline injection (282 vs 165 s). The percentages of using amiodarone (21.43 % vs 57.14 %; *p* value < 0.001), establishment of a definitive airway (35.71 % vs 100 %; *p* value 0.003), and correct detections of pulseless electrical activity (PEA) (28.57 % vs 100 %; *p* value < 0.001) were significantly lower in the online group than the on-scene group. The high quality CPR outcomes between the online group and on-scene group were comparable.

**Conclusions:**

The online medical instruction may have worse CPR outcomes compared with on-scene medical instruction in shockable, simulated CPR scenarios. Further studies are needed to confirm these results.

## Background

Non-traumatic cardiac arrest is a fatal emergency condition [1]. The survival rate of cardiac arrest may increase if it is witnessed and the patient receives basic life support (BLS) within 4 min or advanced cardiac life support (ACLS) within 8 min [2, 3]. Cardiopulmonary resuscitation (CPR) has an aim to have spontaneous cardiac function without post-cardiac arrest brain injury [4]. Both BLS and ACLS including using medications or defibrillations may be needed [4].

CPR can be performed by trained medical personnel or bystanders [4]. A previous study showed that CPR was successfully instructed via telephone [5]. An increase of CPR performance was 11 % after the telephone CPR program was launched; at least four lives were saved [5]. With newer communication technologies, telemedicine such as real time video may be helpful in BLS and chest compression [6, 7]. The advantage of video instruction is real time feedback [7] although some limitations exist such as poor signals or noise during the communication [6]. Telemedicine is also useful in four emergency events such as drowning, burns, intoxication, or renal colicky pain patients during the pre-hospital period in emergency medical services without physicians [8]. There are, however, limited data on the effects of telemedicine in cardiac arrest situations in the literature particularly in Asian settings.

## Methods

### Study design

This study was a retrospective study. Data were retrieved from the 2014 annual Thai national conference in emergency medicine. Two simulated cardiac arrest stations during the conference were studied. These two stations, stations nos. 5 and 11, were a part of the conference activity called “EMS rally” which was comprised of 14 stations. Both of these stations were shockable and out-of-hospital cardiac arrest situations. Station no. 5 was an online instructed station, while station no. 11 was an on-scene station. All teams performed all 14 stations of the rally blinded to others including both online and on-scene instructed stations; similar to a round of OSCE examination. Each team had 11 min in each station. There were 14 representative teams from provinces from all over Thailand participating in the rally. Each team had one physician, one nurse, and two emergency medicine technicians. Physicians acted as an instructor in the scene. Descriptions of the two CPR stations are described below.

Station no. 5 was an out-of-hospital cardiac arrest situation; instructed by the video (VDO) calls. Team members were trained how to use the VDO devices prior to entering the station. Physicians acted as online medical directors and were placed in another room. Physicians were able to order any treatment remotely. The connection between medical directors and team members was performed using Skype®. Two smart phones and two tablets were used during the CPR in this station. One smart phone was connected with the defibrillator screen, while the other one captured the scene of the view from the feet of the patients for overview images. The medical director made any order via the tablets connected with smart phones from the distant center. Team members communicated to others by using bluetooth via smart phones. All connections used the 3G system. The physicians were able to view an overview of the scene, patient status, and electrocardiogram (EKG) waves from the defibrillator, and to make treatment orders.

The scenario for this station was a 58 years old man with history of chest pain and loss of consciousness. The EKG was set to be pulseless ventricular tachycardia (VT) during the first six minutes, pulseless electrical activity (PEA) during the 6^th^–8^th^ minutes, and return of spontaneous circulation (ROSC) until the end of station.

Station 11 had an on-scene medical director. The physician directed the situation onsite with other team members. The scenario was similar to the station no. 5 except the ECG for the first six minutes was ventricular fibrillation instead of VT. The Appendix shows example pictures of both stations.

Both stations were videotaped and outcomes were evaluated for each team. There were eight outcomes measured during the CPR including

1. Time from start to chest compression in seconds.
2. Time from start to VT/ventricular fibrillation (VF) detection correctly in seconds.
3. Time from VT/VF detection to the first defibrillation in seconds.
4. Time from start to adrenaline injection in seconds.
5. Treatment with amiodarone (yes/no).
6. Treatment to establish a definite airway using such as endotracheal tube, laryngeal mask airway, or Esophageal-Tracheal Combitube (yes/no).
7. High-quality CPR defined as three of these following items [6]
  - No interruption of chest compression or chest compression/total time of CPR of more than 80 %.
  - 100–120/min chest compression.
  - Avoidance of excessive ventilation by having assisted respiratory rate less than 12 times/minutes.
8. Correct detection of PEA (yes/no).

### Statistical analyses

The outcomes of the online group were compared with the on-scene group. Time to event outcomes were calculated and presented as median values with 95 % confidence intervals (CI) and Kaplan Meier curves. The Kaplan Meier curves of both groups were compared by the log rank test. Cox regression analysis was used to demonstrate the strength of associations between the outcomes and groups by the hazard ratios. The categorical outcomes are described as numbers and percentages and the differences between groups compared by using the Mc Nemar’s test for dependent proportion comparisons. All statistical analyses were performed by using the STATA software (College Station, Texas, USA).

## Results

There were 14 representative teams who participated in the study that was comprised of a total of 14 physicians, 14 nurses, and 28 emergency medicine technicians. Characteristics of each type of participant are shown in Table 1. The average ages of participants of all three occupations were between the second and third decades of life. The percentage of participants with more than 3 years experience in ambulance medicine was 7.1, 64.3, and 53.6 % in the physicians, nurses, and EMTs groups as shown in Table 1.

**Table 1:** Characteristics of all participants categorized by occupations

Factors	Physicians *n* = 14	Nurses *n* = 14	EMT *n* = 28
Mean age ± SD, years	27.64 ± 2.56	30.64 ± 4.29	29.68 ± 7.013
Age range, years	25–34	26–39	20–49
Gender			
Male	7 (50)	6 (42.9)	23 (82.1)
Experiences in ambulance, years			
< 1 year	6 (42.8)	0 (0)	0 (0)
1–3 years	3 (21.4)	2 (14.3)	8 (28.6)
> 3 years	1 (7.1)	9 (64.3)	15 (53.6)
Not answered	4 (28.6)	3 (21.4)	5 (17.9)
ACLS certified, years			
< 1 year	6 (42.8)	2 (14.3)	5 (17.9)
1–3 years	4 (28.6)	6 (42.9)	10 (35.7)
> 3 years	1 (7.1)	3 (21.4)	3 (10.7)
Not answered	3 (21.4)	3 (21.4)	10 (35.7)
BLS certified, years			
< 1 year	6 (42.8)	1 (7.1)	3 (10.7)
1–3 years	1 (7.1)	3 (21.4)	12 (42.9)
> 3 years	2 (14.3)	4 (28.6)	3 (10.7)
Not answered	5 (35.7)	6 (42.9)	10 (35.7)
Experiences in CPR, year			
< 1 year	3 (21.4)	4 (28.6)	5 (17.9)
1–3 years	4 (28.6)	1 (7.1)	4 (14.3)
> 3 years	3 (21.4)	5 (35.7)	9 (32.1)
Not answered	4 (28.6)	4 (28.6)	10 (35.7)
EMT certified, years	NA	NA	
< 1 year			2 (7.1)
1–3 years			6 (21.4)
> 3 years			3 (10.7)
Not answered			17 (60.7)

Note. Data presented as numbers (percentage) unless indicated otherwise; *EMT* emergency medicine technician, *ACLS* advanced cardiac life support, *BLS* basic cardiac life support, *CPR* cardiopulmonary resuscitation, *NA* not available

**Fig. 1:**
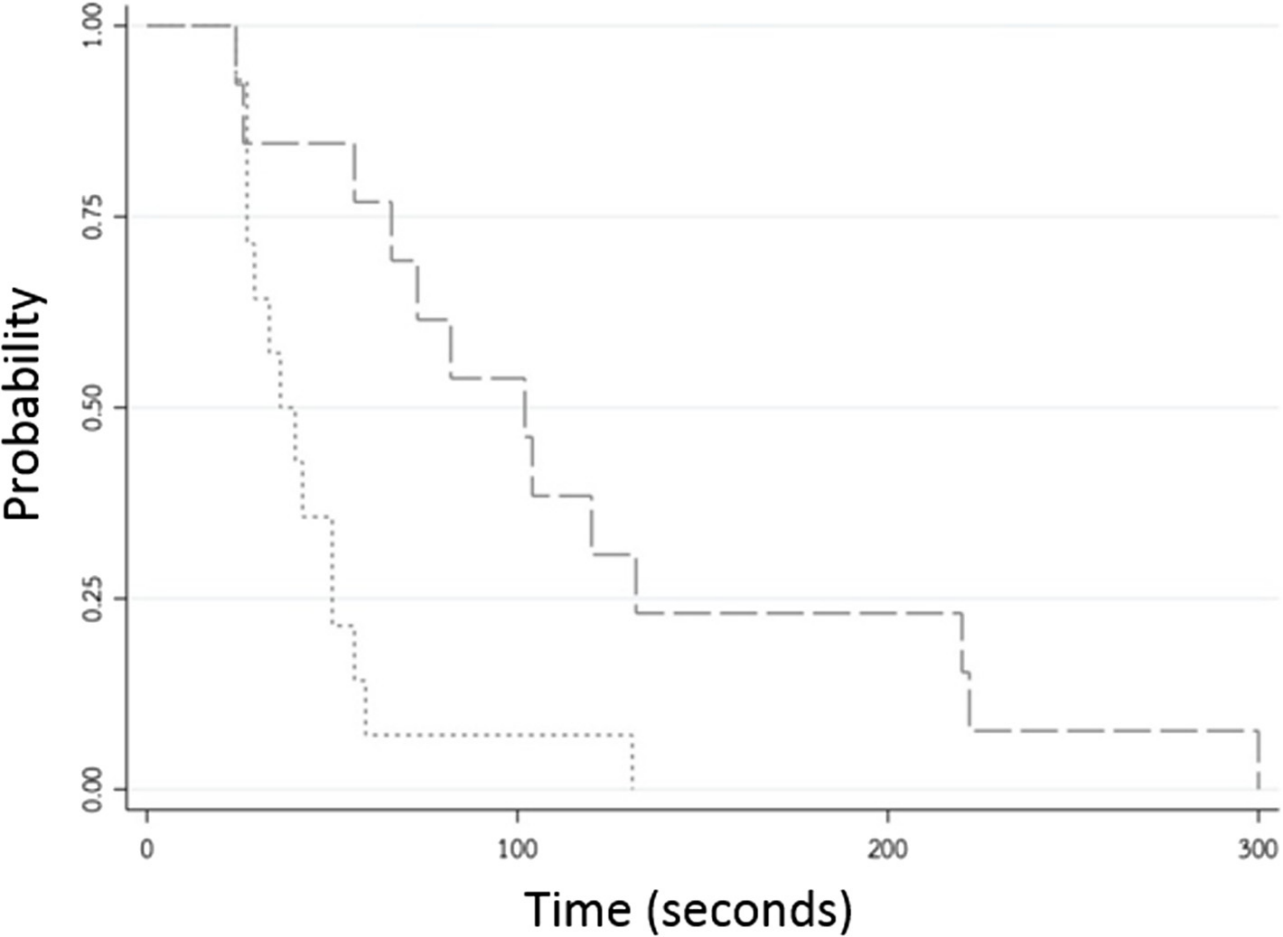
Kaplan-Meier curve of time from start to chest compression between an online medical command (…….) and on-scence medical command (− − −) group

The median times of all outcomes were significantly longer in the online group than on-scene group (Table 2) including total times from start to chest compression (102 vs 36 s), times from start to VT/VF detection (187 vs 99 s); times from VT/VF detection to the first defibrillation (57 vs 28 s); and times from start to adrenaline injection (282 vs 165 s). Kaplan-Meier curves of times from start to chest compressions are shown in Fig. 1.

**Table 2:** Showed median times of CPR outcomes between an online medical command versus on-scene medical command

Outcomes	Median time, seconds		HR	p*
	Online medical command	On-scene medical command		
Time from start to chest compression	102 (95 % CI 56, 132)	36 (95 % CI: 27, 50)	3.98 (95 % CI: 1.61, 9.87)	0.002
Time from start to VT/VF detection	187 (95 % CI: 106, 239)	99 (95 % CI: 77, 128)	9.64 (95 % CI: 2.58, 35.99)	<0.001
Time from VT/VF detection to the first defibrillation	57 (95 % CI: 35, 107)	28 (95 % CI: 15, 39)	2.84 (95%CI: 1.15, 7.01)	0.017
Time from start to adrenaline injection	282 (95 % CI: 226, 390)	165 (95 % CI: 136, 238)	9.81 (95 % CI 2.7, 35,66)	<0.001

Note. *CPR*, cardiopulmonary resuscitation, *HR* hazard ratio by the Cox regression analysis; *VT* ventricular tachycardia, *VF* ventricular fibrillation

*p value of log rank test

The percentages of using amiodarone (21.43 % vs 57.14 %; *p* value < 0.001), establishment of a definitive airway (35.71 % vs 100 %; *p* value 0.003), and correct detections of PEA (28.57 % vs 100 %; *p* value < 0.001) were significantly lower in the online group than in the on-scene group (Table 3). The high quality CPR outcomes between the online group and on-scene group were comparable (Table 3).

**Table 3:** Showed additional CPR outcomes between an online medical command versus on-scene medical command

Factors	Numbers of team		p*
	Online medical command *n* = 14	On-scene medical command *n* = 14	
Amiodarone treatment	3 (21.43 %)	8 (57.14 %)	<0.001
Treatment with definite airway	5 (35.71 %)	14 (100 %)	0.003
No interrupt chest compression	11 (78.57 %)	13 (92.86 %)	0.280
100–120/min chest compression	12 (85.71 %)	13 (92.86 %)	0.541
12/min assisted ventilation	12 (85.71 %)	13 (92.86 %)	0.999
Correctly detection of PEA	4 (28.57 %)	14 (100 %)	<0.001

Note. *CPR* cardiopulmonary resuscitation, *HR* hazard ratio by the Cox regression analysis, *PEA* pulseless electrical activity *p value of Mc Nemar’s test

## Discussion

This study showed that the online medical instruction via VDO call was less effective compared with the on-scene medical instruction for the CPR simulated stations. All outcomes except the factors for the high quality CPR were significantly worse in the online group (Tables 2 and 3). Note that these findings were based on the 14 teams who participated in two simulated shockable scenarios.

Previous studies showed that using video or real time telemedicine may improve CPR procedures [6, 7, 9, 10] particularly in CPR performance. Chest compression rates and depth were better with video instruction compared with the without cell phone video assist station (rates of 95.5 vs 63.0/min and depth of 36.0 vs 25.0 mm; *p* < 0.01 for both factors) [6]. Breathing was also better by adding video from cell phones during CPR [9]. The airways were wider open with the video assist group than the controls (95.3 % vs 58.5 %; *p* < 0.01). There were limited more favorable outcomes of the CPR for the video assisted group. The results of this study indicated that the video assisted CPR may not have favorable outcomes; mainly due to correct detection of PEA (Table 3).

There are several explanations why the online medical command group did not have good CPR outcomes. Participants in the study were informed about the online system and tools only one minute prior to the test station (Station 5). They may not have been familiar with the online system and how to make a decision; this may have delayed the treatment and also assessment of the patient status. One striking finding is the proportion of correct detections of PEA. The online group correctly identified PEA in only five teams (28.57 %), while PEA was correctly detected in all teams with on-scene medical instruction (Table 3). This finding may result in low percentage of airway management and CPR outcomes. Clinical recognition on-scene may be more realistic than the online method. Also, note that a physician was not present in the online method. Communication between the physicians and team members at the scene in the online command group may also be delayed because they needed to communicate via cell phone, while physicians and team members communicated to others immediately in the on-scene group.

The strength of this study is the evaluation of the CPR outcomes in online medical instruction via video calls from the 3G mobile system. These outcomes are limited in the literature which showed only CPR procedures [6, 7, 9, 10]. Some study limitations exist. The study was not a randomized controlled trial due to station rotation sequences. Half of the 14 teams experienced the online type first, while the other half teams experienced the on-scene first. Secondly, the CPR scenarios were limited only to two shockable cases. Thirdly, data regarding CPR experiences were not obtained from all participants. Approximately 70 % of participants, however, provided this information. Finally, the participants were not familiar with the online system. Technical errors and frustration may have occurred resulting in missing or giving the wrong treatment. Further studies with a randomized controlled trial and rigorous online pre-training should be performed.

## Conclusion

Online medical instruction may have worse CPR outcomes compared with on-scene medical instruction in shockable CPR scenarios.

## Abbreviations

ACLS, advanced cardiac life support; BLS, basic life support; CI, confidence intervals; CPR, cardiopulmonary resuscitation; EKG, electrocardiogram; PEA, pulseless electrical activity; ROSC, return of spontaneous circulation; VDO, video; VF, ventricular fibrillation; VT, ventricular tachycardia

## Figures and Tables

**Fig. 2:**
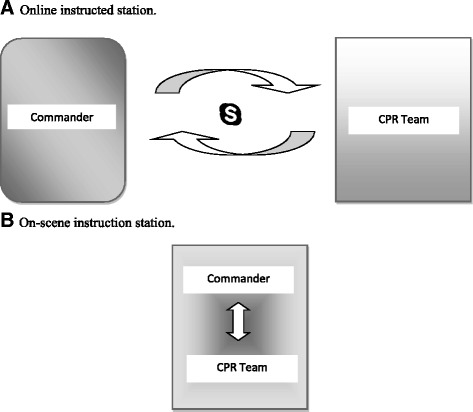
Scheme of the cardiopulmonary resuscitation stations both online (**a**) and on-scene (**b**) instruction
